# Prevalence of low back pain in Iranian nurses: a systematic review and meta-analysis

**DOI:** 10.1186/s12912-017-0243-1

**Published:** 2017-09-11

**Authors:** Yosra Azizpour, Ali Delpisheh, Zahra Montazeri, Kourosh Sayehmiri

**Affiliations:** 1 Department of Clinical Epidemiology, Student Research Committee, Ilam University of Medical Sciences, Ilam, Iran; 2 Department of Clinical Epidemiology, Psychosocial Injuries Research Center, Ilam University of Medical Sciences, Ilam, Iran; 3 School of Epidemiology, Public Health and Preventive Medicine, Faculty of Medicine, University of Ottawa, Ottawa, Canada; 4 Department of Biostatistics, Psychosocial Injuries Research Center, Ilam University of Medical Sciences, Ilam, Iran

**Keywords:** Iran, Low back pain, Nurses, Meta-analysis

## Abstract

**Background:**

Low back pain (LBP) as a musculoskeletal disorder is one of the most common occupational injuries in nurses but there isn’t any valid measure of the prevalence of LBP in Iranian nursing. In order to increase the power and improve the estimates of the prevalence of LBP in Iranian nurses, a comprehensive meta-analysis was carried out. A summary measure of all studies conducted in this field was found and distributions of LBP were evaluated based on different variables.

**Methods:**

Inclusion criteria included articles with prevalence of LBP in Iranian nurses, who had at least six months of work experience without any trauma, injuries to spine, or any underlying disease. The keywords“prevalence, low back pain, nurses”, and “Iran” were used as part of this search. Databases such as Pubmed, Web of Science, Science direct, Scopus, IranMedex, Irandoc, Magiran, SID, CIVILICA, IMEMR and Google scholar were searched up to and including 15 June 2016. For data extraction a form was designed that included the following variables: Author names, province, sample size, age, gender, marital status, work experience, body mass index, job type, smoking status, work schedule, year of publication, type of standard questionnaire, prevalence of LBP, studies’ quality score and climate classifications. Data analysis was carried out using fixed and random effects model. Heterogeneity between studies was assessed by using the I^2^ and Q tests.

**Results:**

In all 1250 articles were identified and 22 articles with 9347 participants met the inclusion criteria for meta-analyses after filtering. The prevalence of low back pain during their working life and during the last year, was estimated at 63% (95% Confidence Interval (CI): 57.4–68.5) and 61.2% (95% CI: 55.7–66.7) respectively. The prevalence rate of this disorder was 58.7% (95% CI: 35.8–81.7) and 60.4% (95% CI: 52.2–68.6) among men and women respectively. Furthermore, prevalence’s of LBP were 59.5% in wards nurses, 50.3% in operating room technicians, and 39.4% in aid nurses.

**Conclusions:**

The results showed the high prevalence of LBP injury in nurses, especially female nurses. The effect of musculoskeletal disorders such as LBP may be reduced by considering proper observation of the principles of ergonomics in the workplace, performing physical examinations on a regular basis, identifying risk factors in the advancement of musculoskeletal disorders and then trying to fix them.

## Background

Musculoskeletal disorders are one of the main issues that arise in the field of health. It is considered globally as the second leading cause of physical disability [1]. The study conducted in Iran that dealt with the burden of disease and injury, and ranked the “disability -adjusted life year” (DALY), indicated that low back pain was in the eighth place among all causes leading to damage including (natural and unnatural) incidences and in fourth place regardless of the latter, [2]. Musculoskeletal disorders are significant problems at work among nurses [3] and LBP is the most important musculoskeletal disorder among them with a rate of 30–60% [4]. Results of a systematic review carried out by Ellapen and Narsigan showed that musculoskeletal disorders in nurses were higher in the lower back, neck, and shoulders [5]. In the studies by Sikiru (Africa) [6] and Freimann (Estonia) [7], 70.87% and 57% of nurses suffered from low back pain during the preceding 12 month period respectively. The Skela-Savič study (Slovenia) indicated that the prevalence of LBP in nurses was 85.9% [8].

We note the negative impact of occupational low back pain. This includes, but is not restricted to, work absence, loss of optimal performance, rising medical costs of treatment and care and occupational disability [9]. Estimating LBP prevalence among nurses is essential for designing control plans and prevention programs. Given that there are no accurate statistics about the prevalence of LBP for nurses in Iran, accordingly, we were encouraged to conduct a study for estimating prevalence of LBP in Iranian nurses.

## Methods

These systematic review and meta-analyses were conducted based on PRISMA guidelines [10].

### Characteristics of studies

All research conducted in the field of LBP prevalence in Iranian nurses, regardless of the publication language (Farsi or English) and time span, were reviewed and included in our study.

### Definitions of variables

Musculoskeletal disorders: Any pain or discomfort in one or more limbs.

Low back pain: Any pain in the lower back between L1 - L5 (lumbar spine) and L5-S1 (lumbosacral joint).

Nurses: Nurses employed in hospitals.

### Data sources

A systematic literature search was conducted in international databases such as Web of Science (1983 to 15 June 2016), Science Direct (1823 to 15 June 2016), PubMed (1966 to 15 June 2016), Scopus (1960 to 15 June 2016), Google Scholar (web search engine), and national databases such as Magiran: an Iranian Journal Database (2001 to 15 June 2016); SID: Scientific Information Database (2000 to 15 June 2016); Iran Medex: an Iranian Biomedical Journal (1982 to 15 June 2016); Irandoc: Iranian Research Institute for Information Science and Technology (1970 to15 June 2016), CIVILICA (Publisher of specialized conferences within the country), (1999 to 15 June 2016); and regional databases including IMEMR: Index Medicus for Eastern Mediterranean Region (1984 to 15 June 2016). The keywords used for this search were “low back pain, musculoskeletal, nurses, prevalence”, and “Iran”. Furthermore, keywords with medical subject headings (MeSh) were used in “advanced searches” in international databases, and these keywords were combined through the use of conjunctions such as OR, AND, NOT. (Equivalents terms in Farsi were used in national databases). It should be noted that articles published in journals and/or conferences, reports, dissertations/theses and all other references to relevant articles were included in our searches.

### Study selection

Inclusion criteria dealt with studies that present prevalence of LBP in nurses in Iran who had at least six months of work experience without any trauma, injuries to spine, or any underlying disease. Exclusion criteria included irrelevant studies, articles without adequate data regarding observations, studies that linked LBP with other diseases, and duplicate studies.

### Data extraction and study quality assessment

Data extraction and study quality assessment was done by two independent researchers (Azizpour and Sayehmiri). Cases of disagreement were solved by a discussion between two reviewers. For data extraction a form was designed that included the following variables: Author names, province, sample size, age (<45 years old and >45 years old), gender (male/female), marital status (single/married), work experience (1–10 year, 11–20 year, 21–30 year), body mass index (underweight <20, normal weight 20–25, overweight 25–30, obese >30), type of job (ward nurse, operating room technicians, and nursing aids), smoking status (yes/no), work schedule (shift work/day work), year of publication, type of standard questionnaire, prevalence of LBP, studies’ quality score. Climate classifications were defined as: 1.Cold climate, 2. Hot and dry climate 3.Temperate and humid climate [11]. Moreover, the adjusted odds ratio and associated confidence interval for variables of gender, work schedule, BMI and work experience was extracted. A modified critical appraisal tool was used to determine the quality and homogeneity of data. This tool includes three methodological tests containing 12 individual criteria for prevalence studies; three questions related to sample representativeness of the target population, six questions related to data quality, and three questions related to the definition of the low back pain disorder (Appendix) [12–14]. Studies with at least 75% of the total score were acceptable.

### Analytical approach

Prevalence of LBP (during the working life and during the last year), in all studies were collected, and then the variance of having LBP was calculated according to the binomial distribution. The weight given to each study was assigned according to the inverse of the variance. Cochrane Q and I^2^ statistics were used to assess heterogeneity among studies. Heterogeneity was measured by I^2^ and divided into four categories; no heterogeneity (0%), low (25–50%), moderate (50–75%), and high (>75%) [15].

In this study we have two effect sizes (ES). The first effect size was prevalence. Due to the heterogeneity of the studies, we computed the prevalence of studies according to a random effects model. The second effect size was the odds ratio. To combine ORs at first we use the log transformation and then we compute Ln OR using the random effect model.

Subgroup analysis and meta-regression (the relationship between the years of the study with the prevalence rate) were employed to explore the cause of heterogeneity between studies. As well, a funnel plot (Begg’s test) with pseudo 95% confidence limits was used to examine publication bias. Data analysis was performed using STATA software version 11 (StataCorp, College Station, TX, USA). Significance level of 0.05 was considered for the *P*-value.

## Results

**Fig. 1:**
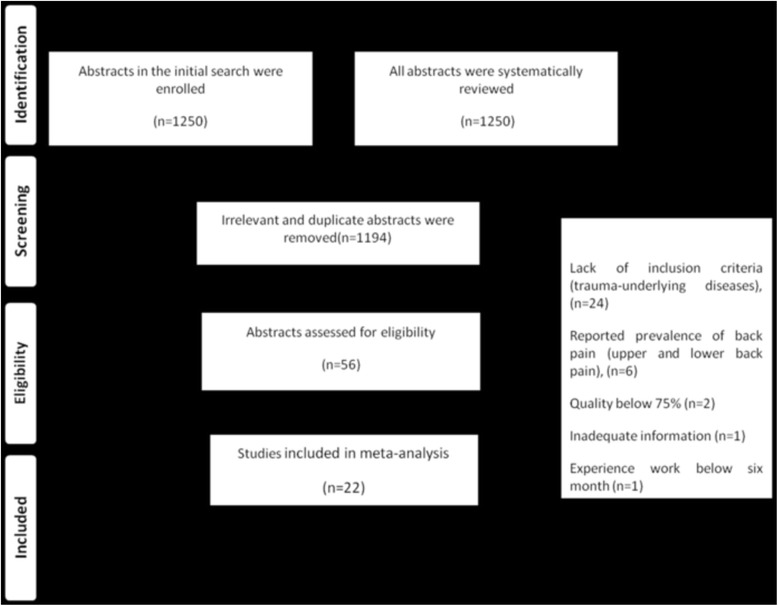
Results of the systematic literature search

In all 1250 articles were identified until 15 June 2016, all abstracts were reviewed and we excluded 1094 irrelevant and 100 duplicate studies. The full texts of the remaining 56 articles were reviewed in detail and finally 22 articles met the inclusion criteria for meta-analyses (Fig. 1). From these the total number of participants was 9347 and twelve studies were written in English (Table 1). All articles were descriptive in nature with a quality score of higher than 80% (Table 2).

**Table 1:** Characteristics of studies included in systematic review and meta-analysis of prevalence of low back pain in Iranian nurses

Author	Province	Sample Size	Gender (%)	Age	Year	Region of Pain	Measures
Mosadeghrad [27]	Isfahan	499	68.5 female	<20–50+ year	2004	LBP	Self-made questionnaire
Sadeghian [28]	Semnan	235	78.3 female	19–50 year	2005	LBP	Nordic Questionnaire
Mohseni-Bandpei [29]	Mazandran	1226	81.9 female	22–57 year	2006	LBP and the sacroiliac joints	Self-made questionnaire
Choobineh [30]	Fars	641	84.7 female	22–66 year	2006	LBP	Nordic Questionnaire
Choobineh [31]	Fars	375	66.4 female	19–62 year	2010	LBP	Nordic Questionnaire
Abedini [32]	Fars	400	89.8 female	30.78±6.44 year	2012	LBP	Nordic Questionnaire
Eftekhar Sadat [33]	Tabriz	195	85.6 female	23–53 year	2013	LBP	Be changed Dutch Questionnaire
Raeisi [22]	Tehran	477	78.4 female	20–60 year	2013	LBP	Nordic Questionnaire
Golabadi [26]	Tehran	545	79.4 female	32.1±7 year	2013	LBP	Nordic Questionnaire
Attarchi [34]	Tehran	454	76 female	20–55 year	2014	LBP	Nordic Questionnaire
Ghasemi [35]	Isfahan	244	68.9 female	N/A	2014	LBP	(VAS) and (ODQ) Questionnaire
Arsalani [36]	Tehran	520	79.4 female	<30–40+ year	2014	LBP	Adapted questionnaire
Pahlevan [37]	Semnan	286	73.5 female	21–52 year	2014	LBP	Nordic Questionnaire
Rezaee [38]	Tehran	1246	53.7 female	20–61 year	2014	LBP	Self-made questionnaire
Zarrin Ghabaee [39]	Mazandran	940	73.6 female	33.7±8.07	2015	LBP	Nordic Questionnaire
Dehdashti [40]	Semnan	48	83.4 female	24–50	2015	LBP	Nordic Questionnaire
Habibi [41]	Isfahan	247	91 female	23–67 year	2015	LBP	Cornell Questionnaire
Azma [42]	Tehran	144	50.7 male	27–43 year	2015	LBP	Cornell Questionnaire
Rokni [43]	Mazandran	110	88.2 female	21–50 year	2016	LBP	Nordic Questionnaire
Taghinejad [44]	Ilam	135	58.5 female	20–59 year	2016	LBP	Nordic Questionnaire
Saremi [45]	Tehran	30	80 female	25–42 year	2016	LBP	Nordic Questionnaire
Asadi [9]	Gilan	350	90.3 female	22–56 year	2016	LBP	Pre-designed checklist

**Table 2:** Investigation of the quality of studies via modified critical appraisal tools

Author	1	2	3	4	5	6	7	8	9	10	11	12	Total Score%
Mosadeghrad [27]	√	√	√	√	√	√	√	NA	NA	√	×	√	90
Sadeghian [28]	√	√	√	√	√	√	√	NA	×	√	√	√	91
Mohseni-Bandpei [29]	√	√	√	√	√	√	×	NA	NA	√	√	√	90
Choobineh [30]	√	√	√	√	√	√	√	NA	NA	√	×	√	90
Choobineh [31]	√	√	√	√	√	√	√	NA	NA	√	×	√	90
Abedini [32]	√	√	√	√	√	√	√	NA	NA	√	×	√	90
Eftekhar Sadat [33]	√	√	√	√	√	√	×	NA	NA	√	√	√	90
Raeisi [22]	√	√	√	√	√	√	√	NA	NA	√	×	√	90
Golabadi [26]	√	√	√	√	√	√	√	NA	NA	√	×	√	90
Attarchi [34]	√	√	√	√	√	√	√	NA	NA	√	×	√	90
Ghasemi [35]	√	√	√	√	√	√	√	NA	√	√	√	√	100
Arsalani [36]	√	√	√	√	√	√	√	NA	NA	√	×	√	90
Pahlevan [37]	√	√	√	√	√	√	√	NA	NA	√	×	√	90
Rezaee [38]	√	√	√	√	√	√	×	NA	NA	√	√	√	90
Zarrin Ghabaee [39]	√	√	√	√	√	√	√	NA	NA	√	×	√	90
Dehdashti [40]	√	√	√	√	√	√	√	NA	NA	√	×	√	90
Habibi [41]	√	√	√	√	√	√	√	NA	√	√	√	√	100
Azma [42]	√	√	√	√	√	√	√	NA	√	√	√	√	100
Rokni [43]	√	√	√	√	√	√	√	NA	NA	√	×	√	90
Taghinejad [44]	√	√	√	√	√	√	√	NA	NA	√	×	√	90
Saremi [45]	√	√	√	√	√	√	√	NA	√	√	√	√	100
Asadi [9]	√	√	√	√	√	√	×	NA	NA	√	×	√	80

### The prevalence of low back pain during a working life

**Fig. 2:**
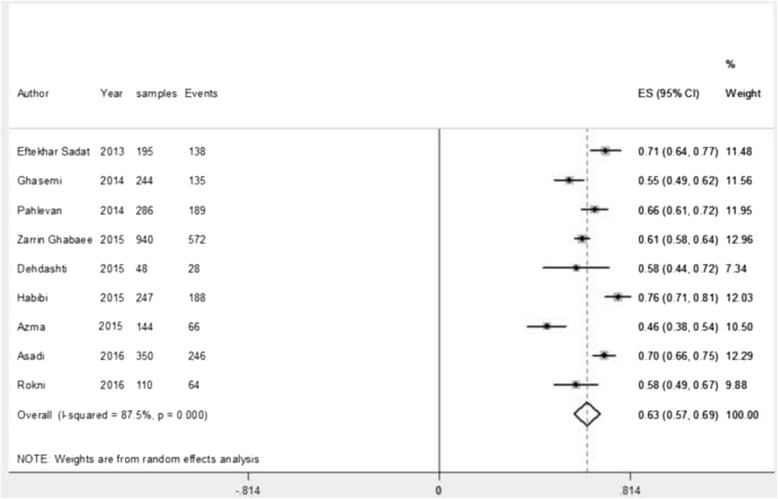
Prevalence of LBP in Iranian nurses during the working life

During the period 2013–2016 nine studies with a sample size of (*n* = 2564) have been carried out. Among them, the lowest and highest prevalence were found to be 45.8% and 76.1%. The estimation of the prevalence rate via the random effects model was found to be 63% (95% CI: 57.4–68.5; *P*-value < 0.0001). Heterogeneity of the reviewed studies was I^2^ = 87.5 and Heterogeneity chi-squared = 64.10 (d.f = 8) *P*-value <0.0001 (highly heterogeneous) (Fig. 2).

### The prevalence of low back pain during the last year

**Fig. 3:**
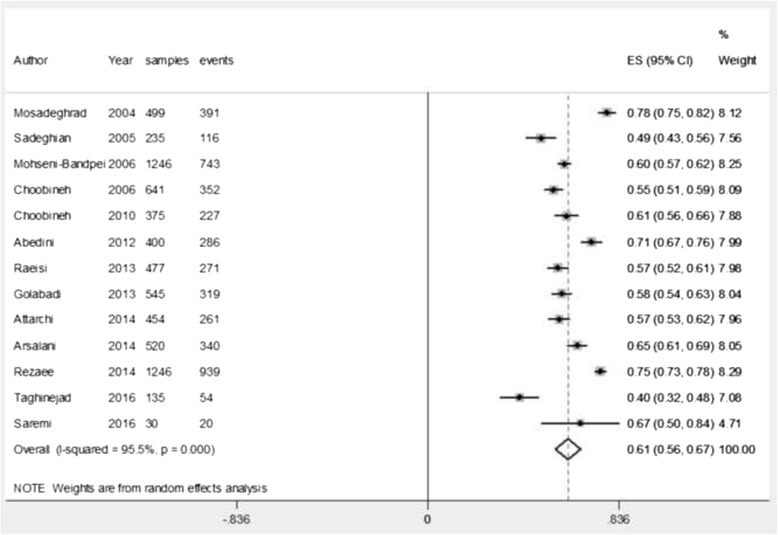
Prevalence of LBP in Iranian nurses during the one last year

During the period 2004–2016, thirteen studies with a sample size of (*n* = 6803) have been carried out. Among them, the lowest and the highest prevalence were found to be 40% and 78.3%. The estimation of the prevalence rate via the random effects model was found to be 61.2% (95% CI: 55.7–66.7; *P*-value < 0.0001). Heterogeneity of the reviewed studies was I^2^ = 95.5 and Heterogeneity chi-squared = 268.37 (d.f = 12) *P*-value < 0.0001 (highly heterogeneous) (Fig. 3).

### Subgroup analysis

According to subgroup analyses, female and married nurses with prevalence rates of 60.4% (95% CI: 52.2–68.6; *P*-value <0.0001) and 74.2% (95% CI: 69–79.4; P-value <0.0001) had the higher prevalence of LBP as compared to their male counterparts, 58.7% (95% CI: 35.8–81.7; P-value <0.0001) and single nurses, 71.3% (95% CI: 67.7–75; P-value <0.0001) respectively. Prevalence of LBP among nurses aged over 45 years, and less than 45 years, were 66% and 54.5% respectively (*P*-value <0.0001). Among nurses with 21–30 years of experience, it was 60% (95% CI: 15–104.9; *P*-value <0.009), but in nurses with experience 1–10 years, the disorder rate was lower (53%). Based on BMI variable, obese nurses and overweight nurses, had prevalence rates of 72.7% (95% CI: 43.7–101.7) and 65.2% (95% CI: 50–80.4) with a higher level of pain in the lower back area as compared to nurses with normal weight and underweight (56.4% and 48.2%), respectively (*P*-value < 0.0001). Prevalence of LBP was 59.5% among ward nurses, 50.3% among operating room technicians, and 39.4% among nursing aids. The rate of LBP was higher among non-smokers, i.e., 73.6% (95% CI: 68.8–78.5; *P*-value <0.0001) in comparison to smokers (Table 3). Furthermore, the prevalence of LBP in a hot /dry climate was 63.7% (95% CI: 56.5–70.8) as well as in the temperate/humid climate, while in a cold climate it was 62.5% (95% CI: 57.7–67.2) and 59.7% (95% CI: 52.3–67.1) respectively.

**Table 3:** Prevalence of low back pain in Iranian nurses according to demographic variables

Variables	Prevalence (%)	Confidence Interval (%)		*P*-value
		lower	upper	
Age				
< 45 year	54.5	44.1	65	0.0001
> 45 year	66	63.7	68.3	0.0001
Gender				
Female	60.4	52.2	68.6	0.0001
Male	58.7	35.8	81.7	0.0001
Marital status				
Single	71.3	67.7	75	0.0001
Married	74.2	69	79.4	0.0001
Work experience				
1–10	53	28.8	77.2	0.0001
11–20	58.3	23.6	93	0.001
21–30	60	15	104.9	0.009
BMI				
< 20	48.2	35.3	61.1	0.0001
20–25	56.4	37	75.8	0.0001
25–30	65.2	50	80.4	0.0001
> 30	72.7	43.7	101.7	0.0001
Nursing job				
Wards nurses	59.5	53.3	65.7	0.0001
Operating room technicians	50.3	39	61.6	0.0001
Aids	39.4	19.9	58.9	0.0001
Smoking status				
Yes	61.1	39.6	82.7	0.0001
No	73.6	68.8	78.5	0.0001

### Effect size of adjusted OR

The risk of LBP in women was 2.44 times more likely than the risk in men (OR = 2.44; 95% CI: 1.89–3.14, *P*-value <0.0001). In nurses with BMI > 25, it was 1.21 times more likely than those with BMI < 25 (OR = 1.21; 95% CI: 0.84–1.74, *P*-value <0.302). In nurses with over 7 years of work experience it was 2.61 times more likely than those nurses with less than 7 years’ experience (OR = 2.61; 95% CI: 2.02–3.37, *P*-value <0.0001). Finally, for those nurses in shift work it was 1.84 times more likely than those involved in day work (OR = 2.44; 95% CI: 1.43–2.37, P-value <0.0001).

### Meta -regression

**Fig. 4:**
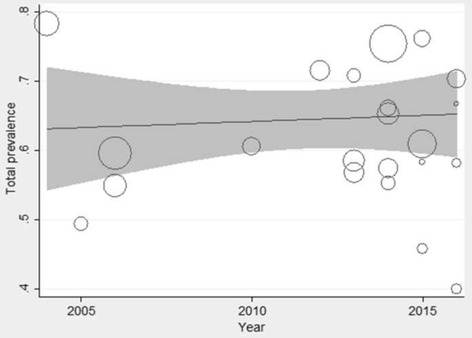
Investigation of the relationship between the year of publication and the prevalence of LBP in Iranian nurses via meta-regression

Meta-regression analysis showed that there was no significant statistical relationship between the year of publication and the prevalence of the LBP (*P*-value = 0.812) (Fig. 4).

### Publication bias

**Fig. 5:**
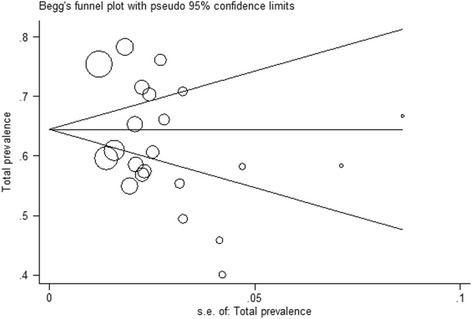
Begg’s funnel plot (pseudo 95% confidence limits) showings significant level of publication bias

Based on the Beggs test, the *p*-value of bias for the studies related to prevalence of low back pain in Iranian nurses is 0.446 (Fig. 5). This identified that the publication bias was not significant.

## Discussion

Overall, the prevalence rate of LBP during a working life and during the previous year was 63% and 61.2% respectively, which indicated a relatively high prevalence. Annual prevalence of LBP in Swiss nurses was found to be 73–76% [16], in comparison with Italian nurses, 86% [17], while in Nigerian nurses it was 44.1% [3], and finally in Japanese nurses it was 71.3% [18] respectively. A comparison of the results revealed that the annual prevalence of LBP among Iranian nurses was lower than Swiss, Japanese and Italian nurses and higher than Nigerian nurses. In nursing practice in many developed countries, due no doubt to the high workloads for patient care, one of the important health concerns is LBP [19].

The results of our study confirmed that the prevalence of LBP in ward nurses is higher than the other groups. In Saudi Arabia, a study conducted on operating room staff revealed that the prevalence of back pain among nurses (anesthesia technician, nurse and operating room technician) was higher than 60% [20], a result that is almost similar to those obtained in our study. The results in Karahan et al. (Turkey) showed that, the prevalence of LBP was 77.1% in nurses, 69.6% in technicians and 53.5% in hospital aides [21]. In another study, Raeisi and colleagues found that the prevalence of LBP in ward nurses was 62.9%, for operating room nurses it found 50%, while in aids nurses it was 49.4% [22]. These results also confirmed the results of our study.

The evidence in this study indicated that prevalence of LBP in nurses over 45 years old with high work experience was higher than any other group. With aging the power of the body (mental and physical) will decrease. In healthcare workers who are also elderly, back pain is a common health problem [23].

The results of this study identified the high prevalence of back pain disorder in women and married nurses in comparison to men and single nurses. As mentioned before the risk of LBP in women was 2.44 times than the corresponding risk in men. It seems factors such as specific physical conditions in women, e.g. lower pain threshold, and physical changes due to monthly menstruation, can be a reason for such disorders in women [24]. In three recent studies married nurses and women nurses in Saudi Arabia [25], single nurses in Taiwan [19], and female nurses in Slovenia [8] had higher rates of low back pain prevalence. In fact, the results of these studies were in agreement with our results with respect to gender, but with respect to marital status, the first study was in agreement whereas the third study was in contrast with ours.

In this study it was observed that demographic factors such as BMI >30 percentile (obesity), work shift (working other than normal hours during a day), gender (women), marital status (married), high work experience, were significantly associated with LBP. However, it was not possible to identify the effects of the physical and mental factors on LBP due to the lack of related information. Golabadi et al. indicated that after adjusting confounding factors among physical demands, awkward position (high status) and static standing posture (high status), psychosocial demands (high status) were highly associated to LBP. Also the nature of the schedule of work (shift work), work experience (>7 years) and gender (female) were significantly associated with prevalence of LBP among nurses [26]. It was shown in another study in Turkey that factors such as age (69.4% in age group 17–24), gender (70% in female), level of education (70.4% in academic level), stand in a day’s work (73.6% >8 h), perception of stress (75.7% in level of very severe) and lifting or carrying heavy objects, had a significant relationship with the occurrence of LBP among hospital staff [21].

Conducting a study at national levels in order to determine psychological and physical stressors in the work environment of nurses and their relationship with musculoskeletal disorders, particularly LBP, to identify the risk factors and to design detailed plans for the prevention and control of these disorders, seems necessary.

### Limitations

The database for gray literature in Iran such as Irandoc was not comprehensive or maybe some researches were done in Iran but they don’t include the results in this database. The structure of report in articles was not same. So that we could not access association some of risk factors with low back pain. For example, in working schedule variable, it was not report in the some articles.

## Conclusion

The results herein showed the high prevalence of low back injury among (Iranian) nurses, especially in female nurses. Proper observation of the principles of ergonomics in the workplace, performing physical examinations on a regular basis, identifying risk factors in the advancement of musculoskeletal disorders, and trying to fix them can be effective factors in reducing musculoskeletal disorders such as back pain among nurses.

## Appendix

**Table 4:** The critical appraisal tool

A: Is the final sample representative of the target population?
1. At least one of the following must apply in the study: an entire target population, randomly selected sample, or sample stated to represent the target population.
2. At least one of the following: reasons for non response described, non responders described, comparison of responders and non responders, or comparison of sample and target population.
3. Response rate and, if applicable, drop-out rate reported.
B: Quality of the data?
4. Were the data primary data of low back pain or was it taken from a survey not specifically designed for that purpose?
5. Were the data collected from each adult directly or were they collected from a proxy?
6. Was the same mode of data collection used for all subjects?
7. At least one of the following in case of questionnaire: a validated questionnaire or at least tested for reproducibility.
8. At least one of the following in the case of an interview: Interview validated, tested for reproducibility, or adequately described and standardized.
9. At least one of the following in the case of an examination: Examination validated, tested for reproducibility, or adequately described and standardized.
C: Definition of low back pain (LBP)
10. Was there a precise anatomic delineation of the lumbar area or reference to an easily obtainable article that contains such specification?
11. Was there further useful specification of the definition of LBP, or question(s) put to study subjects quoted such as the frequency, duration or intensity, and character of the pain. Or was there reference to an easily obtainable article that contains such specification?
12. Were recall periods clearly stated: e.g., 1 week, 1 month or lifetime?
